# Sleep duration and metabolic body size phenotypes among Chinese young workers

**DOI:** 10.3389/fpubh.2022.1017056

**Published:** 2022-10-05

**Authors:** Jiangshui Wang, Dan Xue, Bin Shi, Lu Xia, Weiyi Chen, Li Liu, Junling Liu, Huaiji Wang, Fang Ye

**Affiliations:** ^1^School of Public Health, Tongji Medical College, Huazhong University of Science and Technology, Wuhan, China; ^2^Wuhan Centers for Disease Prevention and Control, Wuhan, China; ^3^Department of Epidemiology and Biostatistics, School of Public Health, Tongji Medical College, Huazhong University of Science and Technology, Wuhan, China; ^4^Department of Occupational and Environmental Health, School of Public Health, Tongji Medical College, Huazhong University of Science and Technology, Wuhan, China; ^5^Key Laboratory of Environment and Health, Ministry of Education and Ministry of Environmental Protection, Wuhan, China; ^6^State Key Laboratory of Environmental Health (Incubating), School of Public Health, Tongji Medical College, Huazhong University of Science and Technology, Wuhan, China

**Keywords:** sleep duration, body size phenotypes, metabolically healthy obesity, young adults, shift work

## Abstract

The evidence linking sleep duration and metabolic body size phenotypes is limited, especially in young adulthood. In this study, we aimed to examine the association between sleep duration and metabolic body size phenotypes among Chinese young workers and investigate whether discrepancies exist among shift and non-shift workers. A cross-sectional study was performed between 2018 and 2019 in Wuhan, China and 7,376 young adults aged 20–35 years were included. Self-reported sleep duration was coded into four groups: <7, 7–8, 8–9, and ≥9 h per day. Participants were classified into four metabolic body size phenotypes according to their body mass index and metabolic health status: metabolically healthy normal weight, metabolically unhealthy normal weight, metabolically healthy overweight/obesity (MHO), and metabolically unhealthy overweight/obesity (MUO). Multinomial logistic regression models were used to explore the associations between sleep duration and metabolic body phenotypes. Compared with those who slept 7–8 h each night, those with sleep duration <7 h per day had higher odds of MHO (OR 1.27, 95% CI: 1.02–1.56) and MUO (OR 1.22, 95% CI: 1.03–1.43), irrespective of multiple confounders. Stratification analyses by shift work showed that the association between short nighttime sleep and increased odds of MUO was only observed in shift workers (OR 1.26, 95% CI 1.03–1.54). Sleep duration is independently associated with metabolic body size phenotypes among Chinese young adults, while shift work could possibly modulate the association. These results may provide evidence for advocating adequate sleep toward favorable metabolic body size phenotypes in young workers.

## Introduction

The prevalence of overweight and obesity is on the rise worldwide, especially in the young population (1). Numerous studies have identified overweight and obesity as a key factor for various cardiometabolic complications, such as type 2 diabetes, hypertension, and hyperlipidemia (2), the morbidities of which are also steadily increasing among young adults (3). However, recent studies proposed that body mass index (BMI) level alone is not enough to distinguish people with different health risks, as a special type of obesity is not always accompanied by cardiometabolic abnormalities (4). To better describe the status of metabolism, the healthier phenotype is called metabolically healthy obesity (MHO), which is characterized by a better metabolic profile, lower inflammation level, more subcutaneous and less visceral adipose tissue (4, 5). In contrast, among normal weight population, there exists a subset exhibiting more cardiometabolic abnormalities, named as metabolically unhealthy normal weight (MUNW) (4). A better understanding of the determinants of these metabolic body size phenotypes is promising to promote relevant effective interventions toward favorable conditions (4, 5).

Factors reported to be associated with obesity and metabolic disorders include unhealthy lifestyles, such as excessive intake of calorically dense foods, lack of physical activity, sleep disorders and occupational factors such as shift work (6–8). Among them, sleep, a fundamental need for humans, attracts more and more attention recently. Previous studies have found that sleep could modulate the development of obesity and metabolic abnormalities through regulating the functioning of daily metabolic and hormonal processes and appetite (9). The US National Sleep Foundation suggested adults aged 18–64 years get 7–9 h of sleep each night (10). For Chinese adults, the recommended sleep duration is 7–8 h per day based on the Healthy China 2030. However, sleep curtailment seems to have become highly prevalent over the past few decades. The percentage of US adults sleeping 6 h or less per night has increased by 31% since 1985 (11), and a large population-based study revealed that 23% of Chinese adults reported getting <6 h of sleep (12). Thus, whether and how inadequate sleep affects metabolic health are needed to be explored.

Although some evidence highlighted the role of inadequate sleep duration in obesity and metabolic indicators (9, 13–17), as far as we're concerned, the association between sleep duration and metabolic body size phenotypes has received scant attention. Previous observational studies were mostly based on children, adolescents (18) or general adults with an average age over 40 years old (19), but with little evidence from the Chinese population or young adulthood. What's more, shift work, related to both insufficient sleep and cardiometabolic diseases (8), was not particularly investigated in previous studies. As sleep duration differs across age groups and short sleep duration and subsequent weight gain seem to be more prevalent in young populations (20), studies targeted at this critical life period are needed and may facilitate pertinent preventive strategies. If effective measures are implemented in early adulthood, corresponding risk factor progression may be reduced or prevented in later life (21). Therefore, we aimed to explore the association between sleep duration and metabolic body size phenotypes among Chinese young adults and investigate whether discrepancies exist among shift and non-shift workers.

## Materials and methods

### Study population

**Figure 1:**
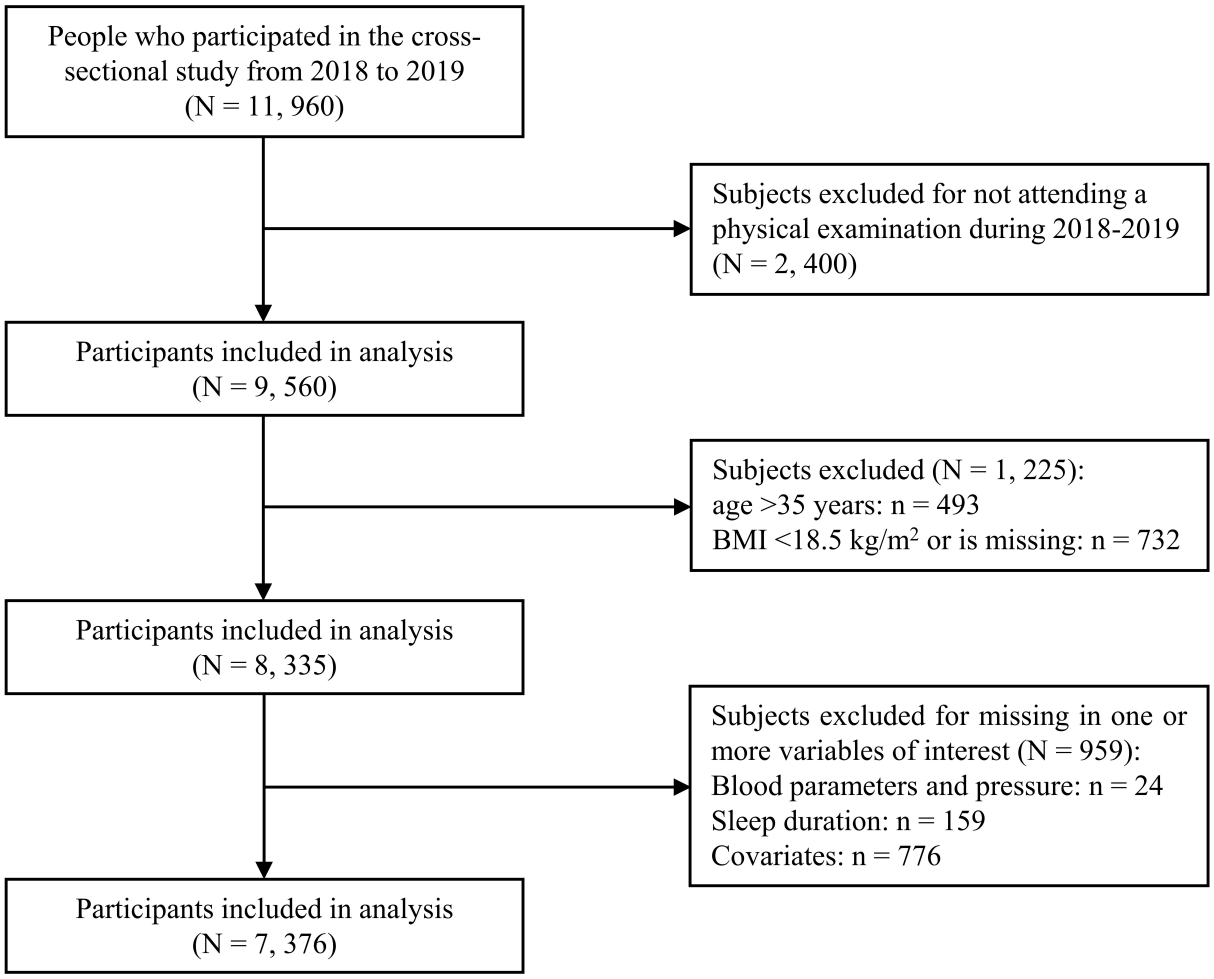
Flow chart of the study participants. BMI, body mass index.

As shown in Figure 1, all employees from Wuhan Metro Group Co., Ltd. were recruited in this cross-sectional study in 2018 and 2019 and 89.2% (11, 960 out of 13, 414) agreed to participate in the current research and finished the questionnaire. For this study, we excluded those who didn't attend a physical examination during 2018–2019 (*n* = 2, 400), those who were older than 35 years old (*n* = 493) (22) and those whose BMI value was missing (*n* = 46) or <18.5 kg/m^2^ (*n* = 686) (23). In addition, we removed those who had missing information regarding variables of interest, such as sleep duration, blood pressure, metabolic parameters and other covariates (*n* = 959). This yielded a final sample size of 7, 376 individuals (6, 095 males, 1, 281 females, with a mean age of 27.1 years).

This study was approved by the Ethics Committee of the Wuhan Centers for Disease Control and Prevention (WHCDCIRB-K-2018042) and written informed consent was obtained from all participants.

### Data collection

All participants were asked to complete a questionnaire and a physical examination. Standardized questionnaires were administered in person by trained interviewers between 2018 and 2019, which included personal characteristics, occupational information, lifestyle behaviors (e.g., smoking, alcohol, sleep, dietary intake and exercise) and medical history. Shift workers were defined as participants with a self-reported history of any work schedule involving irregular working hours instead of a normal daytime work schedule for at least 1 year or else were defined as non-shift workers (24). Smokers were defined as those who had smoked at least one cigarette per day in the past 6 months or else were defined as nonsmokers. Drinkers were considered as those who had drunk alcoholic beverages at least once a week in the past 6 months or else were considered as nondrinkers. Regular exercisers referred to those who have engaged in a variety of physical activities aiming at exercising for more than 20 min per time at least once a week during the past year (25). Similar to a previous study (26), self-reported sleep duration was obtained by asking: “What time do you usually go to sleep at night and wake up in the morning over the past one month?”, and coded into four groups: short (<7 h/d), normal (7–8 h/d), long (8–9 h/d), and very long (≥9 h/d).

Physical examinations were conducted between May 2018 and October 2019 at assigned physical examination centers in Wuhan, Hubei Province. Body weight and standing height were measured with subjects wearing light clothing and no shoes. BMI was calculated as weight/height squared (kg/m^2^). Systolic blood pressure (SBP) and diastolic blood pressure (DBP) were taken in a sitting position after at least 5-min rest. Fasting plasma sample was drawn in the morning after an overnight fast. Concentrations of triglycerides (TG), high-density lipoprotein-cholesterol (HDL-C), and fasting blood glucose (FBG) were measured in the medical centers' laboratories following standard procedures.

### Definition of metabolic body size phenotypes

According to the Working Group on Obesity in China criteria (27), we categorized participants using their BMI into normal weight (18.5–23.9 kg/m^2^), overweight (24.0–27.9 kg/m^2^), obesity (≥28.0 kg/m^2^). Metabolically healthy condition was defined by the current harmonized criteria (28): Participants who met none of the following metabolic abnormalities were considered metabolically healthy: (1) Elevated TG: TG ≥150 mg/dl (1.7 mmol/l), or use of lipid-lowering drugs; (2) Reduced HDL-C: HDL-C < 40 mg/dl (1.0 mmol/l) in men or < 50 mg/dl (1.3 mmol/l) in women, or use of lipid-lowering drugs; (3) Elevated BP: SBP ≥130 mmHg and/or DBP ≥85 mmHg, or use of antihypertensive drugs; (4) Elevated FPG: FPG ≥100 mg/dl (5.6 mmol/l), or use of antidiabetic drugs.

Based on their BMI categories and metabolic health status, participants were classified into four metabolic body size phenotypes: metabolically healthy normal weight (MHNW), metabolically unhealthy normal weight (MUNW), metabolically healthy overweight/obesity (MHO), and metabolically unhealthy overweight/obesity (MUO). The obesity group was combined with the overweight group due to insufficient sample sizes, consistent with previous study (18).

### Statistical analysis

The characteristics of participants were presented as the mean ± standard deviation (SD) or median (interquartile range, IQR) for continuous variables or as number (percentages) for categorical variables across sleep duration groups or the four phenotypes. The analysis of variance (ANOVA) and Chi-square test were performed to compare differences between groups. Subgroup analyses were conducted using Tukey-Kramer's multiple comparisons. Because of the skewed distribution of TG, log-transformed TG was analyzed. Multinomial logistic regression models were performed to examine the associations of sleep duration and metabolic body phenotypes after controlling for various covariates, and further stratified by shift work status to explore whether differences exist among shift and non-shift workers. The reference group was considered as the participants with a sleep duration of 7–8 h/d, which was thought to have the lowest all-cause mortality (29). Covariates were selected based on published literature (19, 26), including age (continuous), gender (male, female), marital status (single, not single), shift work (yes, no), smoker (yes, no), drinker (yes, no), regular exerciser (yes, no) and family history of hypertension (yes, no), diabetes (yes, no) and hyperlipemia (yes, no). Multivariable logistic regression models were then used to further investigate the associations between sleep duration and BMI categories and individual components of metabolic abnormalities. To assess the stability and robustness of our findings, we further adjusted for the consumption of fruit, vegetable, tea and coffee in sensitivity analyses. All statistical analyses were performed using SAS 9.4 software for Windows (SAS Institute Inc., Cary, NC). A two-sided *P* < 0.05 was considered statistically significant.

## Results

### Basic characteristics of participants

Table 1 shows the characteristics of the study population according to sleep duration groups. A total of 7,376 participants were included in this study. The average age of the whole participants was 27.1 years (SD: 2.9). Men made up the majority of the population (6,095, 82.6%). Two-thirds of the participants lived single lives (66.2%). According to their self-reported sleep duration, participants fell into four groups, including <7 h/d (22.0%), 7–8 h/d (31.1%), 8–9 h/d (24.8%), and ≥9 h/d (21.1%). Across four sleep duration groups, there were significant differences in age, gender, marital status, shift work, smoking status, alcohol drinking, physical activity, family history of diabetes, BMI categories, and the levels of TG, HDL-C, and BP (all *P* < 0.05). For example, with the increase of sleep duration, the percentage of smokers gradually declined (<7 h/d: 27.8%, 7–8 h/d: 22.1%, 8–9 h/d: 19.4%, ≥9 h/d: 17.6%, *P* < 0.001). Shift workers accounted for a substantial part of long sleepers (87.2%), followed by 71.4% in the group of 8–9 h/d, 69.5% in the group of < 7 h/d, and 60.2% in the group of 7–8 h/d (*P* < 0.001). As sleep duration increased, more people were of normal weight and fewer were overweight or obese (*P* < 0.001). The sociodemographic characteristics, lifestyles and metabolic indicators were similar between participants included in the present study and those excluded among young adults with a BMI ≥ 18.5 kg/m^2^ who completed the questionnaire and the physical examination during 2018 and 2019 (Supplementary Table S1).

**Table 1:** Basic characteristics of study population according to sleep duration.

**Characteristics**	**Sleep duration, h/d**				
	**<7 (*****N*** = **1,625)**	**7–8 (*****N*** = **2,290)**	**8–9 (*****N*** = **1,832)**	**≥9 (*****N*** = **1,629)**	***P*** ^†^
Age (years, mean ± SD)	27.5 ± 3.0	27.3 ± 2.9	27.1 ± 2.9	26.5 ± 2.7	**<0.001**
Gender (*n*, %)					**<0.001**
Male	1,480 (91.1)	2,058 (89.9)	1,514 (82.6)	1,043 (64.0)	
Female	145 (8.9)	232 (10.1)	318 (17.4)	586 (36.0)	
Marital status (*n*, %)					**<0.001**
Single (unmarried, divorced or widowed)	1,079 (66.4)	1,475 (64.4)	1,162 (63.4)	1,166 (71.6)	
Not single (married or partnered)	546 (33.6)	815 (35.6)	670 (36.6)	463 (28.4)	
Shift worker (*n*, %)	1,130 (69.5)	1,378 (60.2)	1,308 (71.4)	1,421 (87.2)	**<0.001**
Smoker (*n*, %)	452 (27.8)	506 (22.1)	355 (19.4)	286 (17.6)	**<0.001**
Drinker (*n*, %)	332 (20.4)	441 (19.3)	311 (17.0)	249 (15.3)	**<0.001**
Regular exerciser (*n*, %)	667 (41.0)	1,079 (47.1)	927 (50.6)	655 (40.2)	**<0.001**
Family history of hypertension (*n*, %)	392 (24.1)	533 (23.3)	394 (21.5)	379 (23.3)	0.305
Family history of diabetes (*n*, %)	203 (12.5)	237 (10.3)	136 (7.4)	174 (10.7)	**<0.001**
Family history of hyperlipemia (*n*, %)	53 (3.3)	49 (2.1)	38 (2.1)	46 (2.8)	0.070
BMI categories (*n*, %)					**<0.001**
Normal weight (18.5–23.9 kg/m^2^)	838 (51.6)	1,332 (58.2)	1,097 (59.9)	1,090 (66.9)	
Overweight (24.0–27.9 kg/m^2^)	523 (32.2)	700 (30.6)	538 (29.4)	395 (24.2)	
Obesity (≥28.0 kg/m^2^)	264 (16.2)	258 (11.3)	197 (10.8)	144 (8.8)	
TG [mmol/L, median (IQR)]	1.2 (0.8, 1.8)	1.2 (0.8, 1.7)	1.1 (0.8, 1.6)	1.0 (0.7, 1.5)	**<0.001** ^*^
HDL-C (mmol/L, mean ± SD)	1.4 ± 0.3	1.4 ± 0.3	1.4 ± 0.3	1.5 ± 0.3	**<0.001**
SBP (mmHg, mean ± SD)	122.8 ± 13.6	122.4 ± 13.5	122.2 ± 13.4	120.2 ± 13.3	**<0.001**
DBP (mmHg, mean ± SD)	71.9 ± 10.0	71.7 ± 9.7	72.0 ± 9.5	71.2 ± 9.4	0.074
FPG (mmol/L, mean ± SD)	5.1 ± 0.8	5.0 ± 0.6	5.1 ± 0.7	5.0 ± 0.6	0.415

Basic characteristics of study population according to sleep duration are presented as mean ± SD or median (IQR) for continuous variables or as number (percentages) for categorical variables.

BMI, body mass index; TG, triglyceride; HDL-C, high density lipoprotein-cholesterol; BP, blood pressure; FPG, fasting plasma glucose.

† *P*-values were calculated by ANOVA or Chi-square test as appropriate. Values in bold represent statistically significance (*P* < 0.05).

* Log-transformed TG were used to calculate the P-values because of skewed distribution.

### Metabolic characteristics of study population across metabolic body size phenotypes

**Figure 2:**
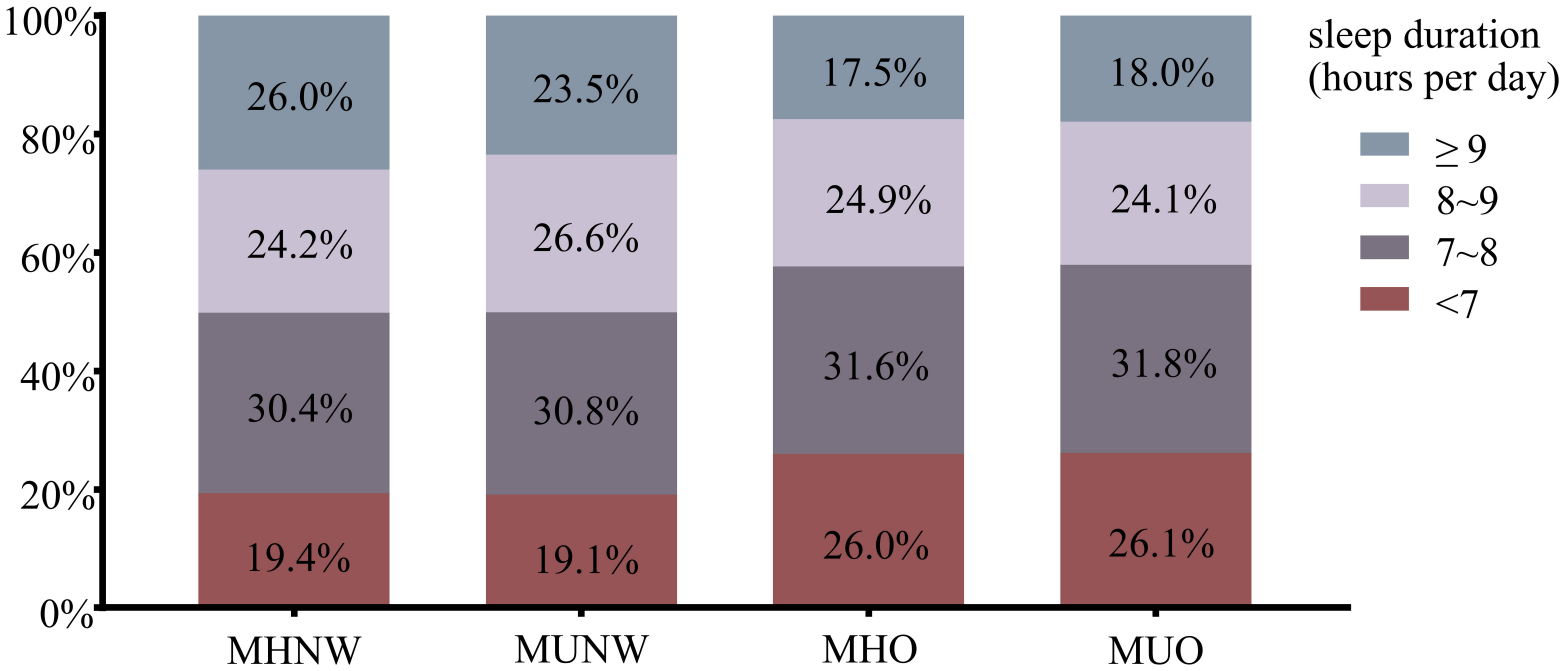
The proportion of sleep duration across metabolic body size phenotypes. MHNW, metabolically healthy normal weight; MUNW, metabolically unhealthy normal weight; MHO, metabolically healthy overweight/obesity; MUO, metabolically unhealthy overweight/obesity.

Table 2 lists the metabolic characteristics of the study population according to metabolic body size phenotypes. The participants were classified into MHNW (35.2%), MUNW (23.9%), MHO (11.9%), and MUO (29.0%). Constituting 29.0% of the overweight/obese individuals, the MHO group presented better metabolic parameters than the MUNW group and the MUO group (lower TG, SBP, DBP and FBG and higher HDL-C), but worse than the MHNW group (higher TG, SBP, DBP, and lower HDL-C) (all *P* < 0.05). The MHO and MUO groups had a higher proportion of individuals with a sleep duration of < 7 h/d (MHO: 26.0%, MUO: 26.1% vs. MHNW: 19.4%) and a lower proportion of those who slept longer than 9 h/d (MHO: 15.5%, MUO: 18.0% vs. MHNW: 26.0%) than those in the MHNW group, as is shown in Figure 2.

**Table 2:** BMI and metaboliccharacteristics across metabolic body size phenotypes.

**Variables**	**MHNW (*N* = 2,596)**	**MUNW (*N* = 1,761)**	**MHO (*N* = 876)**	**MUO (*N* = 2,143)**	***P*** **^†^**
BMI (kg/m^2^)	21.3 ± 1.5^a^	21.7 ± 1.5^b^	26.2 ± 1.9^c^	27.3 ± 2.7^d^	**<0.001**
TG (mmol/L)	0.8 (0.6, 1.1)^a^	1.1 (0.8, 1.8)^b^	1.1 (0.8, 1.4)^c^	1.9 (1.2, 2.6)^d^	<0.001^*^
HDL-C (mmol/L)	1.5 ± 0.3^a^	1.4 ± 0.3^b^	1.4 ± 0.2^c^	1.3 ± 0.2^d^	**<0.001**
SBP (mmHg)	114.1 ± 9.2^a^	126.4 ± 13.7^b^	117.2 ± 8.0^c^	129.7 ± 13.4^d^	**<0.001**
DBP (mmHg)	67.1 ± 6.9^a^	73.9 ± 9.6^b^	68.3 ± 7.0^c^	76.9 ± 10.2^d^	**<0.001**
FPG (mmol/L)	4.9 ± 0.4^a^	5.2 ± 0.6^b^	4.9 ± 0.4^a^	5.2 ± 1.0^c^	**<0.001**

BMI and metabolic characteristics of four body size phenotypes are presented as mean ± SD or median (IQR) as appropriate.

MHNW, metabolically healthy normal weight; MUNW, metabolically unhealthy normal weight; MHO, metabolically healthy overweight/obesity; MUO, metabolically unhealthy overweight/obesity; BMI, body mass index; TG, triglycerides; HDL-C, high density lipoprotein-cholesterol; SBP, systolic blood pressure; DBP, diastolic blood pressure; FPG, fasting plasma glucose.

† *P*-value by ANOVA. Values in bold represent statistically significance (*P* < 0.05).

a, b, c, d Same letters indicate no statistical significance based on Tukey-Kramer's multiple comparison.

* Log-transformed TG were used to calculate the *P*-values because of skewed distribution.

### Association of sleep duration and metabolic body size phenotypes

The multinomial logistic regression analysis was performed to identify the association between sleep duration and metabolic body size phenotypes after adjusting for age, gender, marital status, shift work, smoking status, drinking status, physical activity and family history of hypertension, diabetes and hyperlipemia (Table 3). Compared with those with normal sleep duration (7–8 h/d), those with sleep duration <7 h/d had higher odds of MHO (OR 1.27, 95% CI 1.02–1.56, *P* < 0.05) and MUO (OR 1.22, 95% CI 1.03–1.43, *P* < 0.05), irrespective of confounding factors. ORs with 95% CIs for all variables adjusted for in model 3 are shown in Supplementary Table S2.

**Table 3:** Odds ratios (95% confidence intervals) of metabolic body size phenotypes according to sleep duration categories in multinomial logistic regression models.

**Sleep duration**	**Model 1**	**Model 2**	**Model 3**
**(h/d)**			
**MUNW**			
<7	0.98 (0.82–1.16)	0.97 (0.81–1.16)	0.96 (0.80–1.15)
7–8	Ref	Ref	Ref
8–9	1.08 (0.92–1.28)	1.15 (0.98–1.36)	1.17 (0.99–1.38)
≥9	0.90 (0.76–1.06)	1.10 (0.93–1.31)	1.10 (0.92–1.31)
**MHO**			
<7	1.30 (1.05–1.60)^*^	1.27 (1.03–1.57)^*^	1.27 (1.02–1.56)^*^
7–8	Ref	Ref	Ref
8–9	0.99 (0.80–1.22)	1.09 (0.89–1.34)	1.10 (0.90–1.36)
≥9	0.65 (0.52–0.81)^***^	0.91 (0.72–1.15)	0.92 (0.73–1.16)
**MUO**			
<7	1.29 (1.10–1.51)^**^	1.25 (1.06–1.47)^**^	1.22 (1.03–1.43)^*^
7–8	Ref	Ref	Ref
8–9	0.95 (0.82–1.11)	1.09 (0.93–1.29)	1.11 (0.95–1.31)
≥9	0.66 (0.56–0.78)^***^	1.09 (0.92–1.30)	1.07 (0.89–1.28)

MUNW, metabolically unhealthy normal weight; MHO, metabolically healthy overweight/obesity; MUO, metabolically unhealthy overweight/obesity.

Model 1: unadjusted.

Model 2: adjusted for age and gender.

Model 3: model 2 plus marital status, shift work, smoking status, drinking status, physical activity and family history of hypertension, diabetes and hyperlipemia.

Significance was represented as bold characters,

* *P* < 0.05,

** *P* < 0.01

and

*** *P* < 0.001.

**Figure 3:**
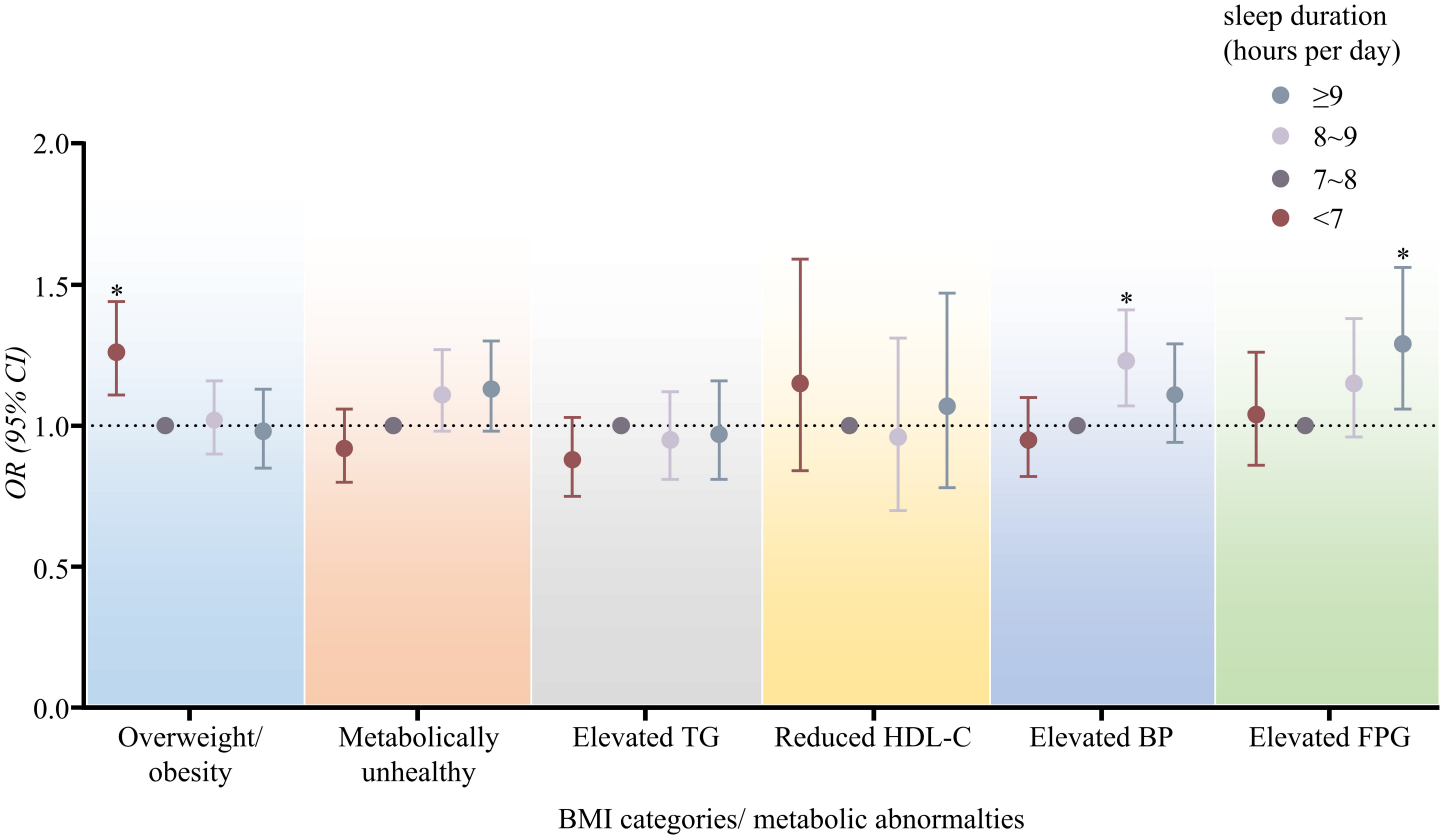
ORs (95% CIs) of BMI categories and metabolic abnormalities according to sleep duration in logistic regression models. OR, odds ratio; CI, confidence interval; TG, triglycerides; HDL-C, high density lipoprotein-cholesterol; BP, blood pressure; FPG, fasting plasma glucose. *P < 0.05. All models were adjusted for age, gender, marital status, shift work, smoking status, drinking status, physical activity. Further adjustment for BMI and family history of hypertension, diabetes or hyperlipemia were made when accessing the association between sleep duration and metabolic abnormalities.

Multivariable logistic regression models were used to further assess the independent effect of sleep duration on BMI categories and metabolic abnormalities, as shown in Figure 3 and Supplementary Table S3. Compared with those who slept 7–8 h/d, those with short sleep duration (<7 h/d) were associated with increased odds of overweight and obesity (OR 1.26, 95% CI 1.11–1.44, *P* < 0.001). Those who slept 8–9 h/d were related to higher blood pressure (OR 1.23, 95% CI 1.07–1.41, *P* < 0.01). Those with nighttime sleep of ≥9 h/d were at higher odds of elevated FPG (OR 1.29, 95% CI 1.06–1.56, *P* < 0.05). Besides, those who slept 8–9 h/d and ≥9 h/d showed a higher tendency toward metabolically unhealthy status (OR 1.11, 95% CI 0.98–1.27 and OR 1.13, 95% CI 0.98–1.30, respectively).

### Association of sleep duration and metabolic body size phenotypes in shift and non-shift workers

Stratification analyses by shift work (Table 4) were also performed. The association between short nighttime sleep and an increased prevalence of MUO was observed only in shift workers (OR 1.26, 95% CI 1.03–1.54, *P* < 0.05), and the relationship between <7 h/d sleep and MHO disappeared (OR 1.24, 95% CI 0.94–1.61, *P* = 0.124). In non-shift workers, we did not find any association between sleep duration and metabolic body size phenotypes.

**Table 4:** Odds ratios (95% confidence intervals) of metabolic body size phenotypes according to sleep duration categories stratified by shift work in multinomial logistic regression models.

**Sleep duration (h/d)**	**Non-shift workers (N** = **2,139)**			**Shift workers (*****N*** = **5,237)**		
	**Model 1**	**Model 2**	**Model 3**	**Model 1**	**Model 2**	**Model 3**
**MUNW**						
<7	0.88 (0.64–1.20)	0.87 (0.64–1.19)	0.87 (0.63–1.19)	1.02 (0.82–1.27)	1.02 (0.82–1.26)	1.00 (0.81–1.25)
7–8	Ref	Ref	Ref	Ref	Ref	Ref
8–9	1.21 (0.91–1.61)	1.23 (0.92–1.63)	1.25 (0.94–1.66)	1.04 (0.85–1.27)	1.13 (0.92–1.38)	1.15 (0.94–1.41)
≥9	0.89 (0.61–1.32)	0.95 (0.64–1.41)	0.96 (0.64–1.42)	0.91 (0.75–1.10)	1.14 (0.93–1.39)	1.14 (0.93–1.39)
**MHO**						
<7	1.45 (1.03–2.04)^*^	1.39 (0.99–1.96)	1.36 (0.97–1.93)	1.25 (0.96–1.63)	1.24 (0.95–1.62)	1.24 (0.94–1.61)
7–8	Ref	Ref	Ref	Ref	Ref	Ref
8–9	0.82 (0.56–1.19)	0.84 (0.58–1.22)	0.85 (0.58–1.24)	1.10 (0.85–1.42)	1.23 (0.95–1.59)	1.25 (0.97–1.62)
≥9	0.55 (0.32–0.96)^*^	0.64 (0.37–1.12)	0.63 (0.36–1.11)	0.70 (0.54–0.91)^**^	1.01 (0.77–1.32)	1.01 (0.77–1.32)
**MUO**						
<7	1.27 (0.96–1.67)	1.17 (0.89–1.55)	1.14 (0.85–1.51)	1.31 (1.08–1.60)^**^	1.29 (1.05–1.58)^*^	1.26 (1.03–1.54)^*^
7–8	Ref	Ref	Ref	Ref	Ref	Ref
8–9	1.06 (0.81–1.39)	1.10 (0.84–1.46)	1.16 (0.88–1.54)	0.92 (0.76–1.12)	1.09 (0.89–1.33)	1.11 (0.91–1.36)
≥9	0.74 (0.51–1.08)	0.91 (0.62–1.35)	0.92 (0.62–1.37)	0.67 (0.55–0.80)^***^	1.14 (0.93–1.39)	1.12 (0.91–1.38)

MUNW, metabolically unhealthy normal weight; MHO, metabolically healthy overweight/obesity; MUO, metabolically unhealthy overweight/obesity.

Model 1: unadjusted.

Model 2: adjusted for age and gender.

Model 3: model 2 plus marital status, smoking status, drinking status, physical activity and family history of hypertension, diabetes and hyperlipemia.

Significance was represented as bold characters,

* *P* < 0.05,

** *P* < 0.01

and

*** *P* < 0.001.

### Sensitivity analyses

We further adjusted for daily consumption of vegetables, fruit, tea and coffee (Supplementary Table S4). The associations of short sleep duration with MHO and MUO were still obtained after adjusting for daily consumption of vegetables and fruit. After we further adjusted for daily consumption of tea and coffee, the relationship between short sleep duration and MHO remained (OR 1.37, 95% CI 1.10–1.70, *P* < 0.01). However, the relationship between short sleep duration and MUO did not reach the threshold of statistical significance (OR 1.16, 95% CI 0.98–1.38, *P* = 0.083).

## Discussion

In the present study, the associations between sleep duration and metabolic body size phenotypes were evaluated among Chinese young workers. We found that compared with normal sleep duration, short nighttime sleep increased the odds of MHO and MUO after adjusting for socioeconomic, lifestyle and disease history confounders. With regard to BMI categories and components of metabolic syndrome, short sleepers appeared to have higher odds of overweight/obesity, while long sleepers had higher odds of elevated blood pressure and fasting plasma glucose. Additionally, the associations between sleep duration and body size phenotypes differed by shift work.

Metabolic body size phenotypes, mutually defined by BMI categories and metabolic health, were proposed in the context where using BMI alone could misclassify people into current health status or potential risks of future clinical outcomes (4, 5, 30). Compared with MUO, an MHO phenotype is characterized by lower liver fat content, greater insulin sensitivity, better insulin secretion and cardiorespiratory fitness (5, 31). Previous research suggested that although people classified as MHO may still bear a higher risk of all-cause mortality and cardiovascular events than MHNW, its risk is substantially lower than MUO (32). In this setting, discovering modifiable factors related to these phenotypes is beneficial for instituting simple lifestyle changes to prevent the development of cardiometabolic diseases, and previous studies have found lifestyles, such as daily exercise and not smoking as potential influencers (5, 32).

To our knowledge, at present there are limited studies examining the association between sleep duration and metabolic body size phenotypes. Ryu et al. found sleep duration different across body size phenotypes with MHO and MUO groups having shorter sleep among Korean general adults (19). Lim et al. found that sleeping <5 h was associated with higher odds of being MHO among Korean children and adolescents (18). Our findings in Chinese young adults are consistent with these results. Although the underlying mechanism of this association is not yet well-elucidated, it is conceivable that the adverse effects of inadequate sleep on obesity and metabolic function could act in this process. Notably, our further analyses about sleep duration with BMI categories and metabolic health showed that short sleep duration was associated with overweight/obesity but not metabolic unhealthy status, which, in line with above studies (18, 19), suggested that excess weight may contribute more than metabolic alterations to the relationship between insufficient sleep and metabolic body size phenotypes. A plausible explanation for it may be that it takes much longer time to develop metabolic alterations than to gain weight (18).

The observed association between short sleep duration and obesity is supported by previous studies (33, 34). A meta-analysis summarized that a reduction of 1 h in sleep per day was associated with an increase of 0.35 kg/m^2^ in BMI among adults (13). Several mechanisms linking short sleep duration with obesity have been proposed. First, it is generally accepted that sleep appears to play a critical role in modulating energy and lipid metabolism in tissues. Population-based studies found that self-reported short sleep duration was associated with decreased leptin levels and increased ghrelin in blood independent of BMI, which could elevate hunger and appetite, resulting in chronically predisposing an individual to overweight or obesity (17). In addition, longer awake time may result in greater fatigue, which tends to decrease physical activity and increase sedentary behavior (35). Sleep-wake cycles also regulate sympathetic nervous system, whose activity gradually decreases during the deep sleep stages of non-REM sleep, while sympathetic nervous activity is elevated during REM sleep and wakefulness stage. Less sleep means less non-REM sleep, resulting in overactivation of sympathetic nervous system, thus a significant reduction of circulating concentrations of catecholamines epinephrine and norepinephrine can be observed in individuals having short sleep duration (36).

In contrast, unanimous agreement has not yet been reached about the effect of sleep duration on metabolic abnormalities. Some studies showed a *U*-shape pattern for sleep duration and higher risks of metabolic syndrome (37), while others confirmed the association only in short sleepers (14, 38) or long sleepers (15), or indicated no significant association between them (33). In the current study, sleep duration was not significantly associated with metabolically unhealthy status after adjustment. However, a trend existed between longer nighttime sleep and metabolically unhealthy status, possibly because long sleep was associated with elevated BP and elevated FPG. Several reasons may contribute to the inconsistency between studies, including different sizes and age structures of the study samples, the fluctuation of BP levels and FPG levels even throughout a day, and unmeasured or unknown confounders.

In stratification analyses, the odds of MUO induced by sleep reduction in groups having shift work were higher in our study, which is important to note given that metro work is a special type of work providing public service for much long time every day and that nearly three-quarters of our participants had shift work experience for at least 1 year. This difference may be caused through effects of shift work on physiological maladaptation to chronically sleeping and eating at abnormal circadian times (8), changes in sleep patterns (24), and decreased secretion of insulin and resting metabolic rate (39). Although further studies are required to better understand the interplays of shift work and sleep with respect to metabolic body size phenotypes, these exploratory results might shed light on finding the key subpopulations in whom adequate sleep duration should be first promoted to prevent cardiometabolic diseases.

Several limitations also need to be considered. First, the cross-sectional design only indicated the association, and the underlying causality cannot be reached. What's more, measurements including sleep duration were self-reported. However, subjective reports of habitual sleep have been shown to be moderately correlated with Actigraphy-measured sleep among adults (40). Third, a single measurement of metabolic parameters might lead to potential misclassification although it's a common practice in large epidemiology studies. Despite these, our study adds new information on the association between sleep duration and metabolic body size phenotypes among young adults.

Taken together, short sleep duration was independently associated with MHO and MUO among Chinese young adults, and its influence appeared to be different depending on shift work. Current results provide scientific evidence for advocating adequate sleep toward favorable metabolic body size phenotypes in young adults.

## Data availability statement

The raw data supporting the conclusions of this article will be made available by the authors, without undue reservation.

## Ethics statement

The studies involving human participants were reviewed and approved by Ethics Committee of the Wuhan Centers for Disease Control and Prevention (WHCDCIRB-K-2018042). The patients/participants provided their written informed consent to participate in this study.

## Author contributions

JW: data curation, conceptualization, methodology, formal analysis, and writing original draft. DX: conceptualization, formal analysis, visualization, and writing original draft. BS and JL: investigation and data curation. LX and WC: data curation and validation. LL: funding acquisition, resources, project administration, and writing—review and editing. HW: project administration and supervision. FY: conceptualization, funding acquisition, resources, supervision, project administration, and writing—review and editing. All authors read and approved the final manuscript.

## Funding

This work was supported by Preventive Medicine Research Project of Hubei Provincial Health Commission-Wuhan Preventive Medicine Research Project (grant numbers WJ2019H303, WJ2019H308). The funders had no role in study design, data collection and analysis, decision to publish, or preparation of the manuscript.

## Conflict of interest

The authors declare that the research was conducted in the absence of any commercial or financial relationships that could be construed as a potential conflict of interest.

## Publisher's note

All claims expressed in this article are solely those of the authors and do not necessarily represent those of their affiliated organizations, or those of the publisher, the editors and the reviewers. Any product that may be evaluated in this article, or claim that may be made by its manufacturer, is not guaranteed or endorsed by the publisher.

## Abbreviations

BMI: body mass index
MHNW: metabolically healthy normal weight
MUNW: metabolically unhealthy normal weight
MHO: metabolically healthy overweight/obesity
MUO: metabolically unhealthy overweight/obesity
BP: blood pressure
SBP: Systolic blood pressure
DBP: diastolic blood pressure
TG: triglycerides
HDL-C: high-density lipoprotein-cholesterol
FPG: fasting plasma glucose
OR: odds ratio
CI: confidence interval.
