# Supplementary material for: Sleep duration and metabolic body size phenotypes among Chinese young workers
Source: Front Public Health. 2022 Oct 5;10:1017056. doi: 10.3389/fpubh.2022.1017056 (PMC9580563; doi:10.3389/fpubh.2022.1017056)
Supplement: Supplementary file 1 [file Data_Sheet_1.docx]

Supplementary Material

**Supplementary Table S1: Characteristics between 7376 participants and 959 non-participants among young adults who attended a physical examination during 2018-2019 with a BMI ≥ 18.5 kg/m^2^.**

**Supplementary Table S2: Odds ratios (95% confidence intervals) of metabolic body size phenotypes according to all variables adjusted for in the multinomial logistic regression model (model 3 in Table 3).**

**Supplementary Table S3: Odds ratios (95% confidence intervals) of BMI categories and metabolic abnormalities according to sleep duration in logistic regression models.**

**Supplementary Table S4: Odds ratios (95% confidence intervals) of metabolic body size phenotypes according to sleep duration categories in multinomial logistic regression models with further adjustment for daily consumption of vegetable, fruit, tea and coffee.**

| **Supplementary Table S1**  Characteristics between 7376 participants and 959 non-participants among young adults who attended a physical examination during 2018-2019 with a BMI ≥ 18.5 kg/m^2^. | | | |
| --- | --- | --- | --- |
| Characteristics | Participants  (N = 7376) | Non-participants  (N = 959) | *P*^†^ |
| Age (years, mean ± SD) | 27.1±2.9 | 27.0±3.0 | 0.292 |
| Gender (n, %) |  |  | **0.021** |
| Male | 6095 (82.6) | 821 (85.6) |  |
| Female | 1281 (17.4) | 138 (14.4) |  |
| Marital status (n, %) |  |  | 0.413 |
| Single (unmarried, divorced or widowed) | 4882 (66.2) | 610 (67.6) ^a^ |  |
| Unsingle | 2494 (33.8) | 293 (32.4) |  |
| Shift worker (n, %) | 5237 (71.0) | 326 (70.9) ^b^ | 0.952 |
| Current smoker (n, %) | 1599 (21.7) | 214 (22.9) ^c^ | 0.381 |
| Current drinker (n, %) | 1333 (18.1) | 175 (18.6) ^d^ | 0.694 |
| Regular exerciser (n, %) | 3328 (45.1) | 403 (42.0) | 0.070 |
| Family history of hypertension (n, %) | 1698 (23.0) | 108 (16.4) ^e^ | **<0.001** |
| Family history of diabetes (n, %) | 750 (10.2) | 42 (6.4) ^f^ | **0.002** |
| Family history of hyperlipemia (n, %) | 186 (2.5) | 10 (1.5) ^g^ | 0.108 |
| BMI categories (n, %) |  |  | 0.674 |
| Normal weight | 4357 (59.1) | 575 (60.0) |  |
| Overweight | 2156 (29.2) | 281 (29.3) |  |
| Obesity | 863 (11.7) | 103 (10.7) |  |
| Metabolic unhealthy (n, %) | 3904 (52.9) | 483 (51.4) ^h^ | 0.371 |
| Elevated TG (n, %) | 1749 (23.7) | 225 (24.0) ^i^ | 0.825 |
| Reduced HDL-C (n, %) | 366 (5.0) | 41 (4.4) ^j^ | 0.437 |
| Elevated BP (n, %) | 2195 (29.8) | 291 (30.4) ^k^ | 0.695 |
| Elevated FPG (n, %) | 1032 (14.0) | 128 (13.7) ^l^ | 0.793 |

BMI, body mass index; TG, triglyceride; HDL-C, high density lipoprotein-cholesterol; BP, blood pressure; FPG, fasting plasma glucose.

^a^ missing for 56, ^b^ missing for 499, ^c^ missing for 26, ^d^ missing for 18, ^e^ missing for 299, ^f^ missing for 299, ^g^ missing for 299, ^h^ missing for 19, ^i^ missing for 23, ^j^ missing for 23, ^k^ missing for 1, ^l^ missing for 23.

^†^ *P* values were calculated by Student's t test or Chi-square test as appropriate.

| **Supplementary Table S2**  Odds ratios (95% confidence intervals) of metabolic body size phenotypes according to all variables adjusted for in the multinomial logistic regression model (model 3 in Table 3). | | | |
| --- | --- | --- | --- |
| Variables | MUNW  (N = 1761) | MHO  (N = 876) | MUO  (N = 2143) |
| Sleep duration (h/d) |  |  |  |
| <7 | 0.96 (0.80~1.15) | **1.27 (1.02~1.56) ^*^** | **1.22 (1.03~1.43) ^*^** |
| 7~8 | ref | ref | ref |
| 8~9 | 1.17 (0.99~1.38) | 1.10 (0.90~1.36) | 1.11 (0.95~1.31) |
| ≥9 | 1.10 (0.92~1.31) | 0.92 (0.73~1.16) | 1.07 (0.89~1.28) |
| Age (years) | 1.02 (0.99~1.05) | **1.06 (1.03~1.10) ^***^** | **1.11 (1.09~1.14) ^***^** |
| Gender |  |  |  |
| Male | ref | ref | ref |
| Female | **0.50 (0.43~0.59) ^***^** | **0.34 (0.27~0.44) ^***^** | **0.16 (0.13~0.19) ^***^** |
| Marital status |  |  |  |
| Single | ref | ref | ref |
| Unsingle | 1.00 (0.85~1.18) | 1.10 (0.90~1.35) | **1.23 (1.05~1.43) ^*^** |
| Shift worker |  |  |  |
| Yes | 1.02 (0.89~1.18) | 0.97 (0.82~1.15) | 1.05 (0.92~1.20) |
| No | ref | ref | ref |
| Current smoker |  |  |  |
| Yes | 0.96 (0.81~1.14) | 1.17 (0.96~1.43) | **1.42 (1.22~1.65) ^***^** |
| No | ref | ref | ref |
| Current drinker |  |  |  |
| Yes | 1.02 (0.86~1.21) | 0.99 (0.80~1.22) | 1.06 (0.90~1.24) |
| No | ref | ref | ref |
| Regular exerciser |  |  |  |
| Yes | 0.89 (0.78~1.00) | 1.10 (0.94~1.29) | 0.93 (0.82~1.05) |
| No | ref | ref | ref |
| Family history of hypertension |  |  |  |
| Yes | **1.29 (1.10~1.51) ^**^** | 1.19 (0.97~1.46) | **1.50 (1.29~1.75) ^***^** |
| No | ref | ref | ref |
| Family history of diabetes |  |  |  |
| Yes | 1.14 (0.91~1.43) | **1.38 (1.05~1.80) ^*^** | **1.42 (1.15~1.76) ^**^** |
| No | ref | ref | ref |
| Family history of hyperlipemia |  |  |  |
| Yes | 0.92 (0.59~1.43) | **1.71 (1.07~2.72) ^*^** | 1.18 (0.79~1.77) |
| No | ref | ref | ref |

MUNW, metabolically unhealthy normal weight; MHO, metabolically healthy overweight/obesity; MUO, metabolically unhealthy overweight/obesity.

Significance was represented as bold characters, ^*^ *P* < 0.05, ^**^ *P* < 0.01 and ^***^ *P* < 0.001.

| **Supplementary Table S3**  Odds ratios (95% confidence intervals) of BMI categories and metabolic abnormalities according to sleep duration in logistic regression models. | | | | |
| --- | --- | --- | --- | --- |
| Variables | Sleep duration, h/d | | | |
|  | <7  (N = 1625) | 7~8  (N = 2290) | 8~9  (N = 1832) | ≥9  (N = 1629) |
| Overweight/Obesity | **1.26 (1.11~1.44) ^***^** | ref | 1.02 (0.90~1.16) | 0.98 (0.85~1.13) |
| Metabolically unhealthy^†^ | 0.92 (0.80~1.06) | ref | 1.11 (0.98~1.27) | 1.13 (0.98~1.30) |
| Elevated TG^†^ | 0.88 (0.75~1.03) | ref | 0.95 (0.81~1.12) | 0.97 (0.81~1.16) |
| Reduce HDL-C^†^ | 1.15 (0.84~1.59) | ref | 0.96 (0.70~1.31) | 1.07 (0.78~1.47) |
| Elevated BP^†^ | 0.95 (0.82~1.10) | ref | **1.23 (1.07~1.41) ^**^** | 1.11 (0.94~1.29) |
| Elevated FPG^†^ | 1.04 (0.86~1.26) | ref | 1.15 (0.96~1.38) | **1.29 (1.06~1.56) ^*^** |

TG, triglycerides; HDL-C, high density lipoprotein-cholesterol; BP, blood pressure; FPG, fasting plasma glucose.

All models were adjusted for age, gender, marital status, shift work, smoking status, drinking status, physical activity.

^†^Further adjusted for BMI and family history of hypertension, diabetes or hyperlipemia when appropriate.

Significance was represented as bold characters, ^*^ *P* < 0.05, ^**^ *P* < 0.01 and ^***^ *P* < 0.001.

**Supplementary Table S4**

Odds ratios (95% confidence intervals) of metabolic body size phenotypes according to sleep duration categories in multinomial logistic regression models with further adjustment for daily consumption of vegetable, fruit, tea and coffee.

| Sleep duration (h/d) | Model 1 | Model 2 | Model 3 | Model 4 (N=7037) | Model 5 (N=6924) |
| --- | --- | --- | --- | --- | --- |
| **MUNW** |  |  |  |  |  |
| <7 | 0.98 (0.82~1.16) | 0.97 (0.81~1.16) | 0.96 (0.80~1.15) | 0.96 (0.80**~**1.15) | 0.99 (0.82~1.19) |
| 7~8 | ref | ref | ref | ref | ref |
| 8~9 | 1.08 (0.92~1.28) | 1.15 (0.98~1.36) | 1.17 (0.99~1.38) | 1.18 (1.00**~**1.39) | 1.18 (1.00~1.40) |
| ≥9 | 0.90 (0.76~1.06) | 1.10 (0.93~1.31) | 1.10 (0.92~1.31) | 1.13 (0.94**~**1.35) | 1.12 (0.94~1.34) |
| **MHO** |  |  |  |  |  |
| <7 | **1.30 (1.05~1.60) ^*^** | **1.27 (1.03~1.57) ^*^** | **1.27 (1.02~1.56) ^*^** | **1.34 (1.08~1.66) ^**^** | **1.37 (1.10~1.70) ^**^** |
| 7~8 | ref | ref | ref | ref | ref |
| 8~9 | 0.99 (0.80~1.22) | 1.09 (0.89~1.34) | 1.10 (0.90~1.36) | 1.14 (0.92**~**1.42) | 1.19 (0.95~1.47) |
| ≥9 | **0.65 (0.52~0.81) ^***^** | 0.91 (0.72~1.15) | 0.92 (0.73~1.16) | 0.93 (0.73**~**1.18) | 0.93 (0.73~1.18) |
| **MUO** |  |  |  |  |  |
| <7 | **1.29 (1.10~1.51) ^**^** | **1.25 (1.06~1.47) ^**^** | **1.22 (1.03~1.43) ^*^** | **1.19 (1.00~1.41) ^*^** | 1.16 (0.98~1.38) |
| 7~8 | ref | ref | ref | ref | ref |
| 8~9 | 0.95 (0.82~1.11) | 1.09 (0.93~1.29) | 1.11 (0.95~1.31) | 1.09 (0.92**~**1.28) | 1.08 (0.91~1.28) |
| ≥9 | **0.66 (0.56~0.78) ^***^** | 1.09 (0.92~1.30) | 1.07 (0.89~1.28) | 1.04 (0.87**~**1.25) | 1.05 (0.87~1.26) |

MUNW, metabolically unhealthy normal weight; MHO, metabolically healthy overweight/obesity; MUO, metabolically unhealthy overweight/obesity.

Model 1: unadjusted.

Model 2: adjusted for age and gender.

Model 3: model 2 plus marital status, shift work, smoking status, drinking status, physical activity and family history of hypertension, diabetes and hyperlipemia.

Model 4: model 3 plus daily vegetable consumption and daily fruit consumption.

Model 5: model 4 plus daily tea consumption and daily coffee consumption.

Significance was represented as bold characters, ^*^ *P* < 0.05, ^**^ *P* < 0.01 and ^***^ *P* < 0.001.
